# Inhibition of Phosphodiesterase 10A by MP‐10 Rescues Behavioral Deficits and Normalizes Microglial Morphology and Synaptic Pruning in A Mouse Model of FOXP1 Syndrome

**DOI:** 10.1002/advs.202500623

**Published:** 2025-06-26

**Authors:** Henning Fröhlich, Jing Wang, Ferdinand Althammer, Tim Schubert, Nina Kluck, Valery Grinevich, Stefanie Schmitteckert, Christian P. Schaaf, Gudrun A. Rappold

**Affiliations:** ^1^ Institute of Human Genetics Heidelberg University 69120 Heidelberg Germany; ^2^ nCounter Core Facility Institute of Human Genetics Heidelberg University 69120 Heidelberg Germany; ^3^ Department of Neuropeptide Research in Psychiatry Central Institute of Mental Health Medical Faculty Mannheim Heidelberg University 69120 Mannheim Germany

**Keywords:** FOXP1 syndrome, mouse model, MP‐10 (PF‐2545920), Pde10a, treatment

## Abstract

FOXP1 syndrome caused by *FOXP1* haploinsufficiency is characterized by intellectual disability, speech, and language impairment, autistic features, and neuropsychiatric abnormalities such as anxiety and hyperactivity. Behavioral changes in patients are mirrored in *Foxp1* ^±^ mice. It is shown that decreased Foxp1 in the *Foxp1* ^±^ striatum results in a significant decrease in phosphodiesterase 10a (Pde10a). Predominantly expressed in medium spiny neurons (MSNs), Pde10a modulates basal ganglia circuitry. Furthermore, the *Foxp1* ^±^ striatum exhibits microglial activation, reduced synaptic pruning, and dysregulation of 111 inflammatory genes. These include the downregulated *P2ry12* and *Fcrls*, markers of homeostatic microglia, and upregulated *Cd74*, a marker of reactive microglia, suggesting that neuroinflammation contributes to the observed deficits. Interestingly, treatment of *Foxp1^±^* mice with the PDE10A antagonist MP‐10 (PF‐2545920) immediately after birth not only corrects behavioral abnormalities, including decreased ultrasonic vocalization, hyperactivity, and anxiety but also normalizes changes in microglia morphology and synaptic pruning. Transcriptomic analysis with a neuroinflammation‐specific gene panel reveals nominal gene expression changes after MP‐10 treatment, including *Bdnf* upregulation and enrichment of neurotrophin signaling. Since FOXP1 and its signaling pathway are highly conserved, administration of MP‐10 or other Pde10a antagonists may also alleviate the neurological dysfunction seen in humans with FOXP1 syndrome.

## Graphical abstract

We demonstrate that *Foxp1* ^±^ mice, modeling *FOXP1* haploinsufficiency, exhibit behavioral deficits, striatal neuroinflammatory changes including altered microglial complexity and synaptic pruning, and markedly reduced Pde10a expression. Pde10a inhibition starting immediately after birth restores *Foxp1* ^±^ behavior, microglial morphology, and pruning, potentially mediated by *Bdnf* upregulation.

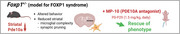

## 1 Introduction

Neurodevelopmental disorders affect around 15% of the population worldwide.^[^ ^1^ ^]^ A key goal of research into neurodevelopmental disorders has always been to develop specific and effective therapies. Each genetically determined phenotype may, however, need to be treated and targeted differently, arguing for the development of stratified drugs for patients. As the etiology of many rare neurodevelopmental disorders is still poorly understood, this is a challenging task. According to Online Mendelian Inheritance in Man (OMIM) (https://www.omim.org/), more than 2800 gene loci are currently associated with isolated intellectual disability (ID) or ID‐associated disorders. A considerable number of the associated genes have been linked with autism spectrum disorders (ASD) and speech and language impairment, including forkhead box protein 1 (FOXP1).

*De novo* haploinsufficiency of *FOXP1* results in a distinct syndrome (MIM 613670), characterized by mild to severe intellectual disability, speech and language impairment, and behavioral abnormalities including ASD or autistic features such as repetitive behavior, anxiety, attention‐deficit/hyperactivity and sensory symptoms.^[^ ^2^, ^3^, ^4^ ^]^ Mitochondrial defects in the striatum and hippocampus contribute to the pathology.^[^ ^5^, ^6^ ^]^ Other clinical findings may include refractive errors, strabismus, cardiac or renal abnormalities, cryptorchidism, hypertonia, hearing loss, epilepsy, and gastrointestinal dysfunction in addition to the known neurological defects.^[^ ^3^, ^4^, ^7^, ^8^ ^]^ However, despite exploring the pathogenesis of FOXP1 syndrome, effective treatments are still lacking. To address this gap in knowledge, this study investigates a possible treatment for FOXP1 syndrome.

FOXP1 belongs to the forkhead box P subfamily of genes, which consists of four members (FOXP1‐4), three of which (FOXP1, FOXP2, and FOXP4) are expressed in the central nervous system.^[^ ^9^, ^10^ ^]^ FOXP1 is highly conserved in vertebrates and expressed in the mouse in both the developing and adult brain, where it exhibits a specific abundance in the projection neurons of the striatum, the cortex (layer III to VIa), hippocampus (CA1 region), and thalamic nuclei.^[^ ^11^, ^12^, ^13^ ^]^ Previous research examining various mouse models has also shown that the striatum is particularly affected by Foxp1 deficiency.^[^ ^14^, ^15^, ^16^, ^17^ ^]^ For example, Nestin‐Cre (*Foxp1* ^‐/‐^) mice completely lack Foxp1 in the nervous system and usually die two to three weeks after birth. In the striatum of embryonic and newborn mice in this line, the enzyme Pde10a was identified as one of 37 significantly dysregulated genes but was not further investigated.^[^ ^7^ ^]^

Pde10a is a dual‐specific phosphodiesterase that inactivates both cAMP and cGMP. It is highly and almost exclusively expressed in the MSNs) of both the direct and indirect output pathways. Because of its function and restricted expression, PDE10A has been considered a potential therapeutic target for various basal ganglia disorders.^[^ ^18^, ^19^, ^20^, ^21^ ^]^ We therefore hypothesized that dysregulation of PDE10A may also play a role in the pathology of FOXP1 syndrome. To test this, we analyzed *Foxp1* ^±^ mice, which display many similar phenotypes to human *FOXP1* haploinsufficiency including behavioral changes reminiscent of ASD.^[^ ^15^ ^]^ All experiments in this study (except Figure S2, Supporting Information), were conducted in *Foxp1* ^±^ animals, which carry a heterozygous deletion of one *Foxp1* allele and thus serve as a valuable mouse model for *FOXP1* haploinsufficiency in humans. In stark contrast to Nestin‐Cre (*Foxp1* ^‐/‐^) mice investigated earlier,^[^ ^7^ ^]^ *Foxp1* ^±^ animals have a normal life expectancy and do not exhibit obvious changes in brain morphology.

In the current study, we investigated whether *Foxp1* ^±^ mice also show an altered Pde10a expression. We tested them for autism‐like behavior, hyperactivity, anxiety, and social behavior in the first 30 days after birth, as cognitive abnormalities manifest in individuals with FOXP1 syndrome in the first 3 years of age. In addition, we investigated to what extent continuous inhibition of Pde10a activity by the specific antagonist MP‐10 (PF‐2545920), a drug with favorable pharmacokinetics and tolerability in clinical trials,^[^ ^22^, ^23^ ^]^ could correct the observed behavioral abnormalities in neonatal and juvenile *Foxp1* ^±^ mice.

The results of our study show that MP‐10 at the administered dose normalized the changes in microglial morphology and synaptic pruning indicative of existing neuroinflammation in the *Foxp1* ^±^ striatum and corrected the existing deficits in early social and anxiety behavior and activity in these animals.

## 2 Results

### 2.1 Striatum of *Foxp1* ^±^ Mice Displays Significantly Reduced Pde10a mRNA and Protein Levels

**Figure 1:**
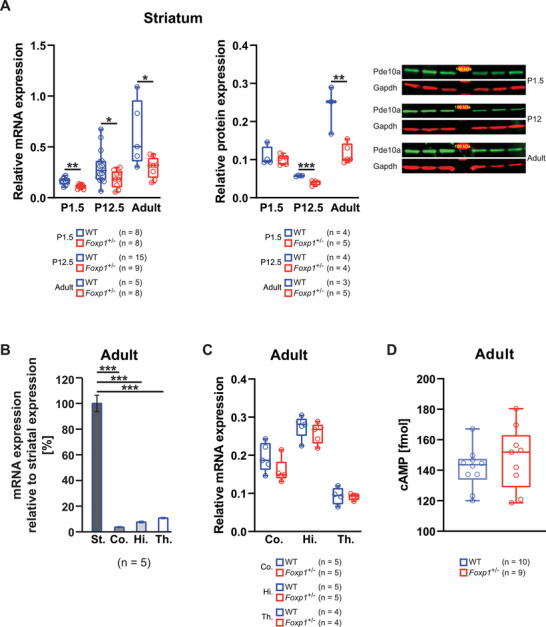
Pde10a mRNA and Protein are Robustly Downregulated in the Striatum of *Foxp1* ^±^ Animals from Birth Through Adulthood. A) Relative expression of *Pde10a* mRNA and protein was compared in striatal tissues from WT and *Foxp1* ^±^ mice at developmental stage P1.5, P12.5 and adulthood by quantitative real‐time PCR and Western blot, respectively. B) Relative Pde10a mRNA expression in adult cortex (Co.), hippocampus (Hi.), and thalamus (Th.) compared with striatum (St.) quantified by real‐time PCR. C) Relative Pde10a mRNA expression in adult WT and *Foxp1* ^±^ cortex, hippocampus, and thalamus quantified by real‐time PCR. D) Quantification of striatal cAMP levels in 7‐month‐old WT and *Foxp1* ^±^ mice. RNA and protein levels in panel A were measured in separate cohorts; each data point represents an individual biological sample. Different animals were used for mRNA and protein analyses. For all box‐and‐whisker plots, the boxes represent the first and third quartiles, the whiskers are the 95% confidence interval, and the lines within the boxes are the median. Asterisks indicate significant difference (^*^ *p* ≤ 0.05, ^**^ *p* ≤ 0.01, ^***^ *p* ≤ 0.001, two‐way ANOVA).

To investigate whether *Foxp1* haploinsufficiency (*Foxp1* ^±^) leads to a reduced Pde10a expression in striatal tissue as previous data of the complete Foxp1 loss in the Nestin‐Cre (*Foxp1* ^‐/‐^) mouse model indicated,^[^ ^7^ ^]^ we examined mRNA and protein expression. Pde10a expression was measured at P1.5, P12.5, and in adult *Foxp1* ^±^ animals by quantitative real‐time PCR and western blot. Both mRNA and protein levels were significantly reduced in *Foxp1* ^±^ striata. In P1 and P12, this reduction amounted to 32% and 40% at the mRNA level and 8% and 32% at the protein level. The strongest effect was seen in adults, where the reduction was 53% at the mRNA level and 51% at the protein level (**Figure** **1**A). In the cortex, hippocampus, and thalamus very low Pde10a expression was detected (Figure 1B) and Pde10a mRNA levels were not significantly reduced in adult *Foxp1* ^±^ animals compared with wild type (WT) (Figure 1C), indicating that Pde10a reduction is striatum‐specific.

Despite significantly reduced Pde10a expression in the striatum of *Foxp1* ^±^ mice, quantification of basal cAMP levels in striatal tissue of 7‐month‐old animals revealed no significant difference compared to WT animals (Figure 1D). This suggests that compensatory mechanisms maintain steady‐state cAMP levels despite reduced Pde10a‐mediated hydrolysis.

### 2.2 Long‐Term Administration of MP‐10 Reverses Behavioral Deficits in Juvenile *Foxp1* ^±^ Animals

**Figure 2:**
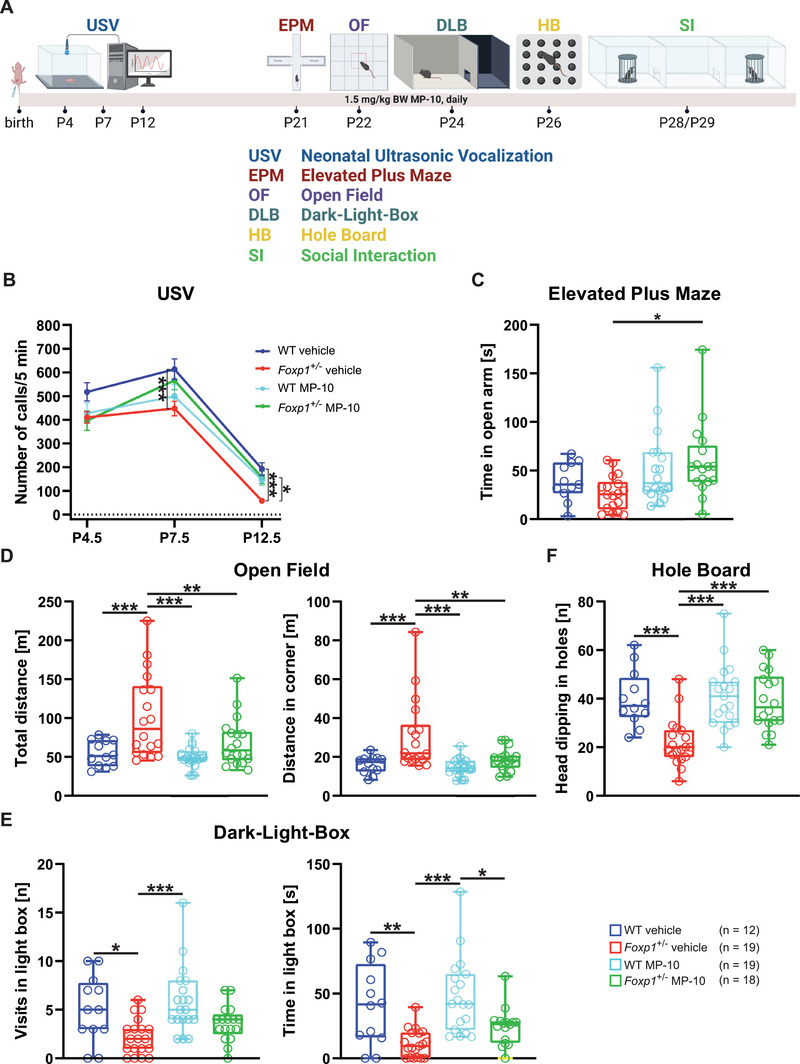
MP‐10 can Reverse Behavioral Deficits in Juvenile *Foxp1* ^±^ Animals. A) Treatment regimen showing the behavioral experiments performed at the respective developmental time points. In all experiments, both vehicle‐injected and MP‐10‐treated WT and *Foxp1* ^±^ mice were examined. B) Isolation‐induced ultrasonic vocalization (USV) recorded at postnatal days 4.5, 7.5, and 12.5. At all three time points *Foxp1* ^±^ mice show a reduced number of calls compared to WT animals. MP‐10 treated *Foxp1* ^±^ pups emit a comparable number of ultrasonic calls as WT pups. C) Elevated plus maze test; *Foxp1* ^±^ mice do not show a significant difference in the time spent in the open arms compared to WT animals. However, MP‐10 treated *Foxp1* ^±^ mice, spend significantly more time in the open arms compared to untreated *Foxp1* ^±^ mice. D) Both the total distance traveled and the distance covered in the corner of the open field are increased compared to WT littermates. *Foxp1* ^±^ animals treated with MP‐10 show no differences in locomotion compared to WT mice. E) Dark‐light‐box test. WT animals enter the light compartment more often than *Foxp1* ^±^ animals and spend more time there. The number of visits to the lightbox and the time spent in the light are not different between MP‐10 treated *Foxp1* ^±^ and WT mice. F) Hole board test; WT animals exhibit a significantly higher number of head dipping into the holes than *Foxp1* ^±^ mice. The number of head dips in MP‐10‐treated *Foxp1* ^±^ animals is comparable to WT mice. Vehicle‐injected WT mice show no significant difference from MP‐10‐treated WT animals in any of the experiments performed. For all box‐and‐whisker plots, the boxes represent the first and third quartiles, the whiskers represent the 95% confidence interval, and the lines within the boxes represent the median. Black asterisks indicate significant differences (^*^ *p* ≤ 0.05, ^**^ *p* ≤ 0.01, ^***^ *p* ≤ 0.001, two‐way ANOVA followed by Bonferroni post hoc test). Figure 2A was created with BioRender.com.

We next addressed the question of whether inhibition of Pde10a in *Foxp1* ^±^ mice could alleviate the behavioral deficits that are associated with *FOXP1* haploinsufficiency. For treatment, we used one of the most advanced PDE10A inhibitors, the highly specific antagonist MP‐10 (PF‐2545920, Mardepodect). This drug exhibits an IC50 of 0.37 nm and >1000‐fold selectivity over other PDEs and has been shown to be safe and well tolerated at doses within the targeted efficacy range.^[^ ^22^, ^23^ ^]^ As reduced Foxp1 levels may already affect brain function at early postnatal stages, treatment of WT and *Foxp1* ^±^ mice was started immediately after birth (at P0.5) and administration of a daily dose of 1.5 mg kg^−1^ MP‐10 or vehicle was continued for 29 consecutive days. The administered dose of MP‐10 was well tolerated with no apparent side effects. During the treatment period, neonatal ultrasonic call behavior, anxiety, and hyperactivity were assessed (**Figure** **2**A). The behavior of treated WT mice did not differ from that of untreated WT mice, and no changes in body weight gain were observed during development. With the exception of the elevated‐plus‐maze test (Figure 2C) and social interaction test, which showed no differences between WT and *Foxp1* ^±^ animals (see Figure S1A–C, Supporting Information), all other tests (neonatal ultrasonic vocalization, open field, hole board, and dark‐light‐box) demonstrated significant behavioral alterations in *Foxp1* ^±^ mice compared with WT animals (Figure 2B,D–F). All performed tests also showed significant behavioral changes between untreated and MP10‐treated *Foxp1* ^±^ animals (Figure 2B–F).

Since Nestin‐Cre (*Foxp1* ^‐/‐^) mice show severely increased anxiety behavior (Figure S2A–D, Supporting Information) and anxiety disorders are common in people with FOXP1 syndrome, we also performed several tests to investigate this further in *Foxp1* ^±^ animals. Here, we show that in the open field test *Foxp1* ^±^ mice traveled 117% more distance compared to WT littermates and covered 91% more distance in the corners, confirming the hyperactivity already described for this test.^[^ ^15^ ^]^ In contrast, MP‐10‐treated *Foxp1* ^±^ animals did not differ from WT in their movement pattern (Figure 2D). Neonatal ultrasonic calling was significantly reduced by approximately 27% in vehicle‐treated *Foxp1* ^±^ pups at P7.5 and P12.5. However, *Foxp1* ^±^ animals receiving MP‐10 showed no difference in the number of calls compared with vehicle‐treated WT littermates (Figure 2B). In the dark‐light box test, both the number of visits to the light and the time spent in this compartment were significantly reduced by 57% and 73% respectively in *Foxp1* ^±^ mice compared to WT controls. In contrast, MP‐10‐treated *Foxp1* ^±^ animals did not show any behavioral changes and behaved like WT animals (Figure 2E). The hole board test also showed aberrant behavior in *Foxp1* ^±^ animals with 45% less head dipping. MP‐10‐treated animals, on the other hand, did not differ from WT animals in this test (Figure 2F).

In conclusion, all behavioral abnormalities analyzed in *Foxp1* ^±^ mice were significantly alleviated by the MP‐10 treatment to the extent that they no longer differed from WT animals in the tests performed.

### 2.3 *Foxp1* ^±^ Mice at P8 Show Signs of Neuroinflammation but no Striatal Atrophy in Adulthood

**Figure 3:**
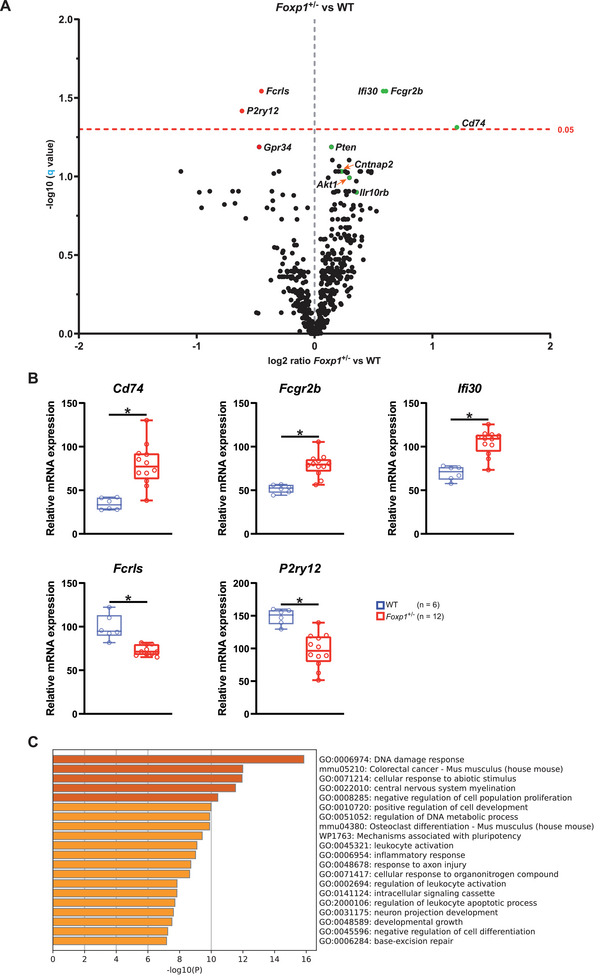
Expression of Neuroinflammatory Marker Genes in the *Foxp1* ^±^ Striatum at P8. To investigate the extent to which there is evidence of neuroinflammation in *Foxp1* ^±^ striatal tissue, a nCounter analysis was performed containing a neuroinflammation panel including 770 targets. A) Volcano plot of differential gene expression between *Foxp1* ^±^ and WT. Genes to the left of the gray vertical dashed line are down‐regulated, and genes to the right are up‐regulated in *Foxp1* ^±^ compared to WT. The marked genes above the dashed red line (*Cd74*, *Fcgr2b*, *Fcrls*, *Ifi30*, and *P2ry12*) show a significant change in expression after correction for multiple tests. They are indicated by either a green dot (if upregulated) or a red dot (if downregulated). The only nominally significantly altered genes in the *Foxp1* ^±^ striatum *Akt1*, *Cntnap2*, *Gpr34*, *Ilr10rb* and *Pten* are also shown and the type of dysregulation is again indicated by the color code. B) Relative mRNA expression of *Cd74*, *Fcgr2b*, *Ifi30*, *Fcrls and P2ry12* in WT and *Foxp1* ^±^ pups as measured by nCounter. C) Analysis of the enriched terms via the input gene list (significant genes after correction for multiple testing and nominally significant genes) using Metascape was performed. A heatmap of the top 20 clusters is shown, colored by p‐values. The lower the p value, the darker the color. For all box‐and‐whisker plots, the boxes represent the first and third quartiles, the whiskers represent the 95% confidence interval, and the lines within the boxes represent the median. Black asterisks indicate significant differences (adjusted p‐value, *Q ≤ 0.05).

Recent evidence points to a contribution of activated glial cells in the pathophysiology of ASD ^[^ ^24^, ^25^ ^]^ and the question arises to what extent neuroinflammation is also present in the *Foxp1* ^±^ striatum. For this reason, bulk expression analysis of WT and *Foxp1* ^±^ striatal tissue at P8 was performed using a NanoString nCounter Neuroinflammation Panel of 770 target genes. A total of 111 genes showed significantly altered expression in the *Foxp1* ^±^ striatum of which 106 genes were nominally significantly altered and five (*Cd74, Fcgr2b, Ifi30, Fcrls*, and *P2ry12*) were significantly altered after correction for multiple testing (see **Figure** **3**A,B; Table S1, Supporting Information). *Cd74, Fcgr2b, Ifi30* are upregulated by ≈ 131%, 52% and 50% respectively, whereas *Fcrls* and *P2ry12* are downregulated by ≈24% and ≈ 35% in the *Foxp1* ^±^ striatum (Figure 3A,B). The nominally significantly altered genes include upregulated *Cntnap2*, as well as upregulated *Pten*, a possible target of Cntnap2, upregulated *Akt1*, downregulated *Gpr34* and upregulated *Il10rb* (see Figure S3 and Table S1, Supporting Information). All ten of the above‐mentioned genes are associated with changes in microglial activity, as detailed in the discussion. Gene Ontology and pathway analysis of the dysregulated genes were performed using Metascape ^[^ ^26^ ^]^ and revealed that the DNA damage response pathway was most significantly affected (Figure 3C; Figure S3B,C, Supporting Information).

As mildly enlarged lateral ventricles, known as *hydrocephalus e vacuo*, have been observed in some individuals with FOXP1 syndrome,^[^ ^27^ ^]^ this raises the question of whether neuroinflammation in *FOXP1* haploinsufficiency can lead to striatal neurodegeneration. Enlarged lateral ventricles can be caused by ventricular defects as well as reduced cortical thickness or atrophy of the striatum. For this reason, we determined the cortical thickness and volume of lateral ventricles in adult WT and *Foxp1* ^±^ mice using MRI (Figure S4A–C, Supporting Information). Our results show that neither the cortical thickness (Figure S4B, Supporting Information) nor the volume of the lateral ventricles (Figure S4C, Supporting Information) is altered in *Foxp1* ^±^ brains compared to WT brains, which clearly argues against striatal atrophy.

### 2.4 MP‐10 Modulates Neurotrophin and Immune Signaling Pathways

**Figure 4:**
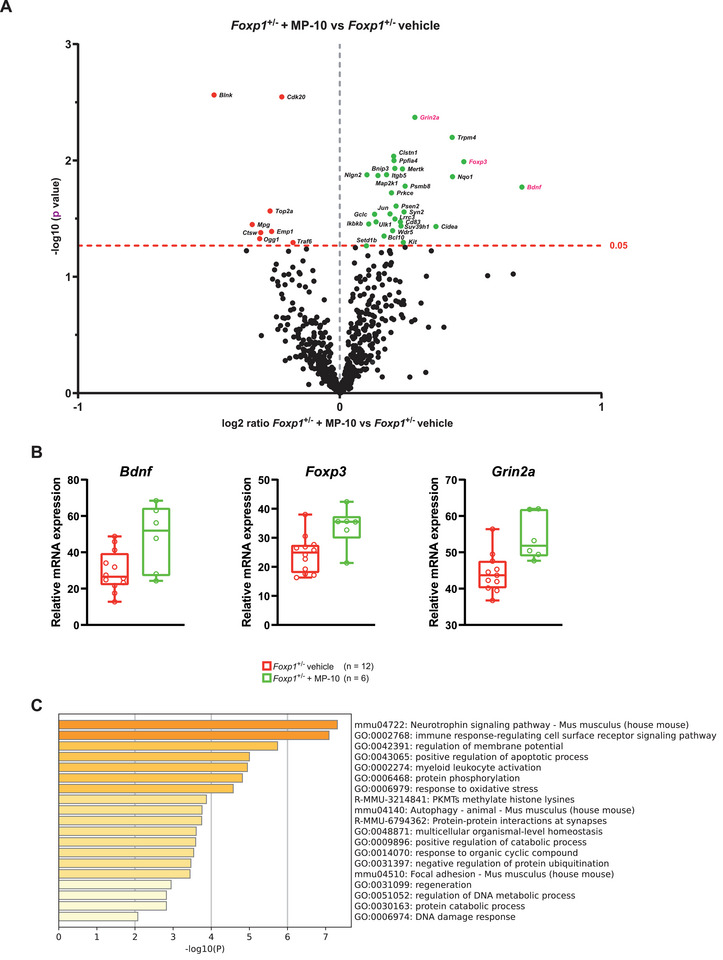
Influence of MP‐10 on Neuroinflammatory Marker Gene Expression in the *Foxp1* ^±^ Striatum at P8. To investigate the influence of Pde10a inhibition on the expression of neuroinflammatory genes in the *Foxp1* ^±^ striatum, *Foxp1* ^±^ animals were treated with vehicle or MP‐10 immediately after birth until P8 and a nCounter analysis was performed with the previously used neuroinflammation panel. A) Volcano plot of differential gene expression between MP‐10 and vehicle‐treated *Foxp1* ^±^ mice. The marked genes above the dashed red show a nominal significant change in expression. Genes to the left of the gray vertical dashed line are down‐regulated, genes to the right are up‐regulated in MP‐10 treated compared to vehicle‐treated *Foxp1* ^±^ mice. *Bdnf*, *Foxp3* and *Grin2a*, genes of particular interest, are highlighted in pink. B) Relative mRNA expression of *Bdnf* and Bdnf regulated *Grin2a* and *Foxp3* in WT and *Foxp1* ^±^ pups as measured by nCounter. C) Analysis of the enriched terms via the input gene list (nominally significant genes) using Metascape was performed. A heatmap of the top 20 clusters is shown, colored by p‐values. The lower the p‐ value, the darker the color. For all box‐and‐whisker plots, the boxes represent the first and third quartiles, the whiskers represent the 95% confidence interval, and the lines within the boxes represent the median.

To investigate the effect of MP‐10 treatment on the expression of neuroinflammatory markers in the striatum, we analyzed *Foxp1* ^±^ pups at P8 in which treatment was started immediately after birth and continued daily as before. Thirty‐six genes showed nominally significant differences (P < 0.05) between MP‐10‐treated and untreated *Foxp1* ^±^ mice (**Figure** **4**A; Table S1, Supporting Information). As none remained significant after correction for multiple tests, these results are exploratory and hypothesis‐generating. Notably, *Bdnf* and the Bdnf‐regulated genes *Grin2a* and *Foxp3* were upregulated by 62%, 22%, and 39%, respectively (Figure 4B), potentially contributing to the observed reduction in microglial activation. Pathway analysis of the nominally regulated genes suggests that MP‐10 administration affects neurotrophin signaling and the immune response‐regulating cell surface receptor signaling pathway (Figure 4C; Figure S5, Supporting Information).

### 2.5 MP‐10 Administration Normalizes Striatal Microglia Activity and Reduces Microglia‐Mediated Synapse Elimination in the *Foxp1* ^±^ Brain

**Figure 5:**
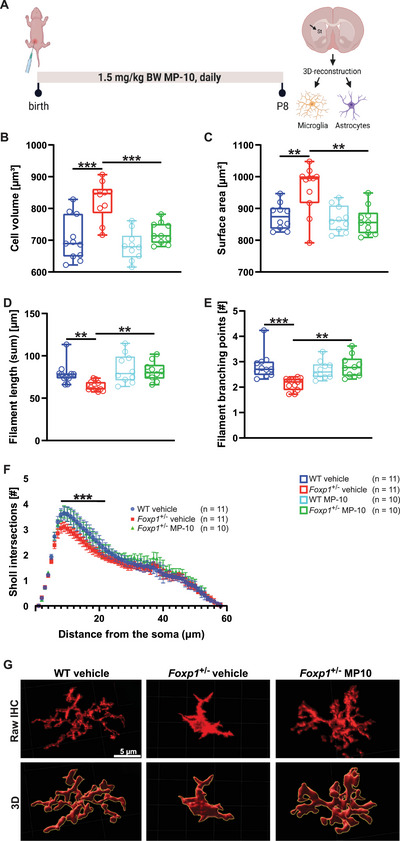
*Foxp1* ^±^ Mice Show Altered Microglial Morphology in the Striatum at P8, Which can be Corrected by MP‐10 Treatment. A) Treatment scheme; WT and *Foxp1* ^±^ animals were treated daily with either vehicle or MP‐10 immediately after birth, and striatal microglia and astrocyte morphology was subsequently assessed by 3D reconstruction with Imaris at P8. Microglia and astrocytes were labeled by immunofluorescent antibody staining for Iba1 and GFAP, respectively. BW, body weight; St, striatum. B–F) Microglia from vehicle‐treated *Foxp1* ^±^ animals show increased cell volume (B) and surface area (C), whereas filament length (D) and filament branching (E, F) are significantly reduced compared to vehicle‐treated WT animals. MP‐10‐treated *Foxp1* ^±^ animals show normal microglial morphology. G) Representative microglia (red) before (RAW IHC) and after 3D reconstruction (3D) of WT and vehicle‐ or MP‐10‐treated *Foxp1* ^±^ tissue. Astrocyte morphology does not differ between the genotypes and is therefore not shown in this figure. For all box‐and‐whisker plots, the boxes represent the first and third quartiles, the whiskers represent the 95% confidence interval, and the lines within the boxes represent the median. Black asterisks indicate significant differences (^**^ *p* ≤ 0.01, ^***^ *p* ≤ 0.001, two‐sided *t*‐test). Figure 5A was created with BioRender.com.

We have shown above that neuroinflammatory processes in the *Foxp1* ^±^ striatum can be modulated by MP‐10 administration. As there is increasing evidence that glia cell alterations play a role in neurodevelopmental disorders such as ID and ASD,^[^ ^24^, ^25^, ^28^ ^]^ we examined both striatal astrocytes and microglia in WT and *Foxp1* ^±^ animals. We compared microglia morphology by 3D reconstruction at P8 in vehicle‐ and MP‐10‐treated WT and *Foxp1* ^±^ pups. To this end, we labeled microglia by immunofluorescence antibody staining against ionized calcium‐binding adaptor molecule 1 (Iba1) and analyzed microglial morphology and complexity using Imaris software. A total of 42 animals were analyzed (n = 10–11 per group), with approximately 1500 microglia reconstructed and analyzed per animal. As neuroinflammatory processes are characterized by a complex interplay between microglia and astrocytes, we also labeled the latter by immunofluorescence antibody staining of glial fibrillary acidic protein (GFAP) to assess the GFAP intensity as a potential readout of astrogliosis/astrocyte hypertrophy in the *Foxp1* ^±^ striatum. No differences were detected between WT and *Foxp1* ^±^ astrocytes (Figure S6A, Supporting Information), and the number of microglia did not differ between genotypes (Figure S6B, Supporting Information). In contrast to astrocytes, *Foxp1* ^±^ microglia exhibited a 17% increase in cell volume and a 10% increase in area compared to WT microglia (**Figure** **5**A,B). In addition, filament length and branching were reduced by 19% and 25%, respectively (Figure 5C–E), indicative of a pro‐inflammatory microglial phenotype.^[^ ^29^ ^]^ MP‐10‐treated *Foxp1* ^±^ animals, however, showed no difference in microglial cell volume, area, filament length, and branching compared with vehicle‐treated WT animals (Figure 5A–F).

To investigate whether this effect was region‐specific, we also examined microglial morphology in the hippocampus. Similar to the striatum, *Foxp1* ^±^ hippocampal microglia exhibited a pronounced 45% increase in somatic cell volume and 32% decrease in filament length compared to WT controls, indicative of a reactive microglial phenotype. However, in contrast to the striatum, MP‐10 treatment failed to normalize microglial morphology in the hippocampus, with somatic swelling persisting in MP‐10‐treated *Foxp1* ^±^ animals (see Figure S7, Supporting Information).

**Figure 6:**
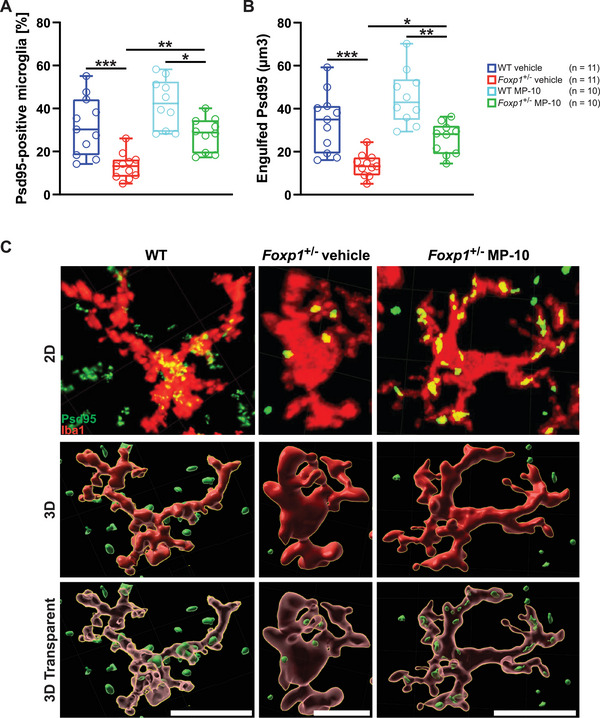
Synaptic Pruning is Significantly Increased in the Striatum of Untreated *Foxp1* ^±^ Mice at P8, and Rescued in MP‐10‐Treated *Foxp1* ^±^ Animals. To examine synaptic pruning in those mice that were treated daily with vehicle or MP‐10 immediately from birth to P8, microglia, and synapses were labeled by fluorescent antibody staining for Iba1(red) and Psd95 (green), respectively. A,B) Both the number of synapse engulfing microglia and the amount of engulfed synaptic material are significantly increased in *Foxp1* ^±^ striata. *Foxp1* ^±^ animals treated with MP‐10 show no differences in synaptic pruning compared to WT animals. C) Representative microglia (red) with synapse (green) before (RAW IHC) and after 3D reconstruction (3D) of WT and vehicle or MP‐10 treated *Foxp1* ^±^ tissue. For all box‐and‐whisker plots, the boxes represent the first and third quartiles, the whiskers represent the 95% confidence interval, and the lines within the boxes represent the median. Black asterisks indicate significant differences (^*^ *p* ≤ 0.05, ^**^ *p* ≤ 0.01, ^***^ *p* ≤ 0.001, two‐sided *t*‐test (A–E) and two‐way ANOVA (F).

Given that microglia‐mediated synaptic pruning is a critical process in shaping neuronal circuits during development and disease, we further investigated synaptic elimination in the striatum. Synaptic contacts and engulfment were assessed by co‐labeling microglia and the postsynaptic marker PSD‐95. In the *Foxp1* ^±^ striatum, microglia exhibited a 59% reduction in synaptic contacts and engulfed 59% fewer synapses compared to WT (**Figure** **6**A–C). Strikingly, MP‐10 treatment restored both the number of synaptic contacts and the level of engulfment in *Foxp1*⁺/⁻ striata to levels comparable with WT tissue (Figure 6A–C).

In summary, *Foxp1* haploinsufficiency results in altered microglial morphology and reduced microglia‐mediated synapse elimination, highlighting the contribution of neuroinflammation to the pathological phenotype. Importantly, early MP‐10 treatment effectively prevents both microglial activation and impaired synaptic pruning in the striatum, but not in the hippocampus.

## 3 Discussion

Our study shows that reduced Foxp1 expression in the *Foxp1* ^±^ striatum is accompanied by greatly reduced Pde10a mRNA and protein levels during development and into adulthood, while the cortex, hippocampus, and thalamus are not affected. We can also demonstrate that continuous administration of the Pde10 antagonist MP‐10 from birth can completely reverse early deficits in social behavior, anxiety, and hyperactivity, as well as changes in striatal microglia and synaptic pruning.

It is well known that Pde10a modulates the signaling of the cyclic nucleotides cAMP and cGMP. Highly and almost exclusively expressed in striatal MSNs, Pde10a plays a critical role in regulating striatal activity and basal ganglia circuitry.^[^ ^30^, ^31^ ^]^ Blocking PDE10A with specific antagonists has previously reduced symptoms of Huntington's disease (HD) and Parkinsons's disease (PD) in mice, rats, and monkeys, although PDE10A expression is reduced in both disorders.^[^ ^32^, ^33^, ^34^ ^]^ We now also show reduced Pde10a expression in *Foxp1* ^±^ animals. Although reduced Pde10a expression would be expected to increase intracellular cAMP levels, our measurements in bulk striatal tissue showed no significant difference in basal cAMP concentrations between adult *Foxp1* ^±^ and WT animals. This may be explained by functional redundancy with other striatal phosphodiesterases, subcellular compartmentalization of cAMP signaling,^[^ ^30^, ^35^ ^]^ or the fact that Pde10a's role becomes more apparent under conditions of synaptic activity and dopaminergic stimulation.^[^ ^36^ ^]^ Moreover, it remains unclear whether the loss of one *Foxp1* allele affects Pde10a expression equally in D1‐ and D2‐MSNs, which could further influence local signaling dynamics.

Although counterintuitive, inhibition of this phosphodiesterase has a positive effect on symptoms, as PDE10A antagonists act similarly to D2 receptor blockers by increasing the activity of MSNs of type D2 (D2‐MSN) of the indirect (striatopallidal) pathway.^[^ ^37^, ^38^, ^39^, ^40^ ^]^ Due to a lower cAMP threshold in D1‐type MSNs of the direct pathway (D1‐MSNs) compared with D2‐MSNs, antagonism of Pde10a may result in more pronounced downstream effects in D2‐MSNs, ultimately leading to a balance between the direct and indirect pathways.^[^ ^39^ ^]^

In this context, it is interesting to note that *Foxp1* ^±^ mice exhibit hyperexcitability of D2‐MSNs, whereas no change was detectable in D1‐MSNs.^[^ ^15^, ^41^ ^]^ Increased intrinsic excitability could also be confirmed in D2‐MSNs with homozygous *Foxp1* deletion. This was primarily caused by the downregulation of two classes of potassium currents: inward rectifying currents (K_IR_) and leak currents (K_Leak_). The downregulation of both Kcnj2 and Kcnj4 and the downregulation of Kcnk2 detected in these neurons may underlie the K_IR_ and K_Leak_ currents respectively.^[^ ^41^ ^]^ As Pde10a is known to regulate intracellular signaling in striatal MSNs and exerts strong control over gene expression,^[^ ^42^, ^43^ ^]^ altered Pde10a expression may be responsible for the downregulation of these potassium channels.

We have demonstrated that *Foxp1^±^* mice exhibit alterations in striatal microglial morphology and reduced synaptic pruning in pups. Microglia, the resident immune cells of the central nervous system, constantly scan their local microenvironment and sense impairments triggered by endogenous and/or exogenous factors. In pathological conditions, microglia are activated, and the resulting dysregulation of certain genes is thought to be an inevitable part of almost all CNS pathologies.^[^ ^44^ ^]^ Reduced complexity and surface area in *Foxp1* ^±^ microglia indicate activation. Microglial activation is characterized by a transformation from a branched morphology with small cell bodies and highly branched, filamentous cell processes to a morphology with a large cell body and short, stout, unbranched cell processes, as has been described in early brain development.^[^ ^45^ ^]^

It is well known that in pathological conditions, such as neuroinflammation, microglia lose their transcriptomic homeostatic signature. The altered expression of 111 neuroinflammatory genes, in particular the significantly increased expression of *Cd74*, *Fcgr2b*, and *Ifi30*, and the downregulation of *Fcrls* and *P2ry12* in the striatum of *Foxp1* ^±^ pups supports this finding. *P2ry12* is one of the most commonly used markers to distinguish microglia from other macrophages. Its reduced expression is considered one of the salient features of the microglial response to Alzheimer's pathology and other disease conditions.^[^ ^46^ ^]^ Upon activation, the expression of P2RY6 is increased, while that of P2RY12 is decreased, indicating a functional shift from chemotaxis to phagocytic function.^[^ ^47^, ^48^ ^]^ Another homeostatic marker that is downregulated during activation is *Fcrls*, which is reduced by 27% in *Foxp1* ^±^ mice. Microglial activation is also confirmed by the significant upregulation of *Cd74* which is considered a marker for reactive microglia.^[^ ^49^ ^]^ Moreover, it is well known that Cd74 mediates the binding of the extracellular pro‐inflammatory cytokine macrophage migration inhibitory factor, which is released in response to stress or an inflammatory response.^[^ ^50^ ^]^ Increased expression of Fcgr2b as found in the *Foxp1* ^±^ striatum appears to play a crucial role in PD, as it binds aggregated α‐synuclein and thus inhibits phagocytosis of microglia through SHP‐1 activation.^[^ ^51^ ^]^ It is possible that this mechanism also contributes to the observed reduction of synaptic pruning in *Foxp1* ^±^ microglia. Ifi30 is known to play an important role in neurodegenerative processes.^[^ ^52^ ^]^

Inflammatory processes triggered by the subsequent activation of microglia suggest that neuronal cell adhesion molecules (CAMs), such as Cntnap2, may play a role in inflammatory cascades. *Cntnap2*, which is downregulated in the *Foxp1* ^±^ striatum, has been implicated in various neurodevelopmental disorders including autism.^[^ ^53^ ^]^ Another point in favor of neuroinflammation is the upregulation of *Akt* and *Pten* and the downregulation of *Gpr34* in the *Foxp1* ^±^ striatum. Dysfunctional PI3KAKT signaling may contribute to the development and maintenance of neuroinflammation as it is closely linked to microglial activity and activation. Abnormalities in this pathway can lead to a pro‐inflammatory microglial phenotype that contributes to a variety of diseases and disorders.^[^ ^54^ ^]^ Microglia are known to have high constitutive expression of the Il‐10 receptor, suggesting that they may be subject to recurrent activation that must be controlled by the anti‐inflammatory Il‐10.^[^ ^55^ ^]^ The increased *Il10rb* expression detected in *Foxp1* ^±^ tissue may therefore be a compensatory mechanism due to existing neuroinflammation.

Treatment with MP‐10 normalized the altered striatal microglial morphology and reduced synaptic pruning in *Foxp1* ^±^ pups. Interestingly, MP‐10 administration increased mRNA levels of neurotrophic factor (Bdnf), and neurotrophin signaling was found to be the most affected pathway in treated versus untreated *Foxp1* ^±^ striatum. Although gene expression changes reported here are based on nominal P‐values without correction for multiple testing and are therefore considered to be exploratory, they provide valuable hypotheses for future targeted analyses. Bdnf acts as an important regulator of neuronal development, survival, and plasticity and is necessary for neuronal and functional maintenance in the striatum. By supporting survival at their origin, Bdnf determines the size of the striatum promotes the maturation of striatal neurons, and facilitates the establishment of striatal connections during brain development. In addition, it is known to have a significant anti‐inflammatory effect and BDNF signaling in microglia has been shown to play crucial physiological roles in learning and memory by promoting learning‐related synapse formation.^[^ ^56^ ^]^

Our results thus support the anti‐inflammatory effect of Pde10a inhibition by suppression of both microglial activation and proinflammatory gene expression.^[^ ^18^, ^19^ ^]^ Increased neurotrophin signaling after MP‐10 administration may also contribute to the behavioral improvements and normalization of microglia morphology and synaptic pruning in *Foxp1* ^±^ animals. Interestingly, the effects of MP‐10 on microglial morphology appear to be brain region‐specific. While microglial activation in the striatum was fully normalized by MP‐10 treatment, this was not the case in the hippocampus. Here, *Foxp1* ^±^ animals also exhibited a pronounced increase in microglial somatic volume and decreased filament length, consistent with a reactive phenotype. However, hippocampal microglia in MP‐10‐treated *Foxp1* ^±^ animals remained hypertrophic and failed to revert to a WT‐like morphology. Notably, in contrast to the striatum, no reduction in *Pde10a* levels was observed in the *Foxp1* ^±^ hippocampus. This finding suggests that the anti‐inflammatory effects of Pde10a inhibition may be restricted to a brain region with high endogenous Pde10a expression, the striatum, or that region‐specific differences in microglial responsiveness to cAMP/cGMP modulation exist. Given the known regional heterogeneity of microglial phenotypes and their interaction with local neuronal and astroglial signals, these results emphasize the need for a more nuanced understanding of how Pde10a antagonism affects glial function across different brain areas.

There is already strong evidence for the contribution of glial cells to the pathophysiology of ASD. Resting microglial cells are of relevance in regulating learning and memory, including modulation of memory strength, forgetfulness, and memory quality, through mediation of synaptic pruning. In response to neuroinflammation, microglia are activated and secrete proteins such as cytokines, chemokines, and reactive oxygen species. Through their dynamic morphological and functional properties, they influence synaptic function and plasticity.^[^ ^57^ ^]^ Moreover, glial cell function is associated with an imbalance between excitatory and inhibitory synaptic functions.^[^ ^58^ ^]^ Indeed, brain samples from autistic individuals showed gliosis and increased glial proliferation,^[^ ^25^ ^]^ and animal models of ASD such as Rett syndrome, Fragile X syndrome, and a mouse model of tuberous sclerosis revealed glial abnormalities. Of note, we recently demonstrated mitochondrial dysfunction and increased oxidative stress in the *Foxp1* ^±^ striatum and hippocampus.^[^ ^5^, ^6^ ^]^ Mitochondrial DNA is considered an important activator of inflammation and can lead to inflammasome activation when it escapes from stressed mitochondria.^[^ ^59^, ^60^ ^]^ For this reason, it is likely that mitochondrial dysfunction in the *Foxp1* ^±^ brain contributes to or is even causative for the development of the observed microglial abnormalities. Further studies should clarify whether the improvements in behavior after MP‐10 administration are due to an effect of the compound on mitochondrial function and accumulation of reactive oxygen species (ROS) in MSNs and possibly microglia.

Alterations in cortical‐subcortical circuits have been shown to affect motor function, cognition, and emotional behavior. Dysfunction of corticostriatal circuits and imbalance of both direct and indirect basal ganglia pathways serve as the neurobiological basis for a myriad of disorders. These disorders include, but are not limited to, neurodegenerative diseases like HD and PD, psychiatric disorders like schizophrenia (SCZ), developmental disorders like ASD, and various anxiety disorders.^[^ ^61^ ^]^ Moreover, it is well established that movement disorders and altered basal ganglia circuits in HD, PD, and SCZ are associated with changes in PDE10A expression and function.^[^ ^21^ ^]^ In addition, mutations in *PDE10A* itself cause childhood‐onset chorea with striatal lesions.^[^ ^62^ ^]^ Therefore, this molecule is considered a potential therapeutic target for these aforementioned disorders, as well as other basal ganglia disorders.^[^ ^21^, ^63^, ^64^, ^65^, ^66^, ^67^ ^]^ Indeed, autism‐like behaviors induced in rats by administration of valproic acid or a serotonin receptor agonist were significantly attenuated by treatment with papaverine, a PDE10A antagonist.^[^ ^66^ ^]^ The same treatment also had a positive effect on neuroinflammatory processes and oxidative stress. However, compared to MP‐10, a potent, orally active, and selective PDE10A inhibitor with an IC50 of 0.37 nm, with >1000‐fold selectivity over other PDEs, papaverine has significantly lower potency and selectivity and a very short exposure half‐life after systemic administration.^[^ ^23^ ^]^

It is interesting to note that both HD mouse models and HD patients have reduced levels of PDE10A as well as reduced levels of FOXP1.^[^ ^68^, ^69^, ^70^ ^]^ Given that Pde10a is a Foxp1 target and is decreased in both Nestin‐Cre (*Foxp1* ^‐/‐^) and *Foxp1* ^±^ mice, it is reasonable to hypothesize that reduced Foxp1 expression may also be responsible for the reduced striatal Pde10a levels in HD. Unfortunately, phase II clinical trials with MP‐10 (NCT01806896, NCT02197130, NCT02342548) in patients with advanced symptoms of HD failed to meet the prespecified endpoints of the trial, despite the promising data in HD mouse models.^[^ ^33^, ^34^ ^]^This result, however, cannot be extrapolated to FOXP1 syndrome for multiple reasons. First, the time point of therapeutic intervention may be crucial for success. It is increasingly understood that therapeutic interventions during sensitive periods in brain development, typically before the onset of symptoms, will affect prospective results. PDE10A expression has been described as altered well before the symptomatic onset in HD.^[^ ^71^ ^]^ This suggests that at the clinical onset, many neurons are already affected which might flag them for destruction. Thus, the dysregulated PDE10A‐mediated intracellular signaling may represent an early phenomenon in this late‐onset disorder that is irreparable at the time of clinical diagnosis. Second, in cortical neurons expressing mutant htt, elevated Foxp1 expression protected these cells from cell death,^[^ ^68^ ^]^ whereas knockdown of Foxp1 in healthy neurons promoted cell death.^[^ ^68^ ^]^ These findings convincingly show that FOXP1 plays a neuroprotective role in striatal and cortical neurons and that reduced FOXP1 expression causes the selective neurodegeneration of striatal and cortical neurons in HD brains.^[^ ^68^ ^]^ In contrast, MRI analysis of adult *Foxp1* ^±^ mice revealed no changes in striatal or cortical size, and there is no evidence of striatal neurodegeneration in humans with *FOXP1* haploinsufficiency.^[^ ^27^, ^72^ ^]^ *Foxp1* ^±^ animals show a decrease in *Foxp1* mRNA and protein of ≈ 41% and ≈ 52%, respectively, at P1, P12, and adulthood compared to WT animals.^[^ ^5^ ^]^ However, significantly lower FOXP1 levels were observed in both a HD mouse model and HD patients.^[^ ^71^ ^]^ It is therefore conceivable that FOXP1 levels gradually decrease with age in HD patients and that MSNs become apoptotic when FOXP1 expression falls below a certain threshold.

To date, more than 650 individuals with a pathogenic variant in FOXP1 have been documented, but this number is expected to increase due to genetic testing. As the FOXP1 transcription factor and its regulatory networks are highly conserved,^[^ ^73^ ^]^ it is likely that reduced PDE10A expression is a major contributor to the symptomatology not only in mice but also in humans with FOXP1 syndrome. As the 4‐week treatment period in newborn mice is equivalent to more than a decade of life in children, further studies can be performed to examine whether the start of treatment also at later developmental time points remains beneficial and if inhibition of Pde10a by MP‐10 still ameliorates learning and memory deficits during later development.

Most people with FOXP1 syndrome have autistic traits and behavioral problems such as hyperactivity, attention problems, impulsivity, aggression, anxiety, mood instability, obsessions, and compulsions. Moreover, many suffer from attention‐deficit/hyperactivity disorder, often in combination with hyperactivity and inattention. The effect of the antipsychotics administered is largely mediated by blocking the postsynaptic dopamine D2 receptors. Aripiprazole (a partial D2 receptor antagonist) and risperidone (a D2 receptor antagonist) are the most commonly used antipsychotics and the only medications approved by the US Food and Drug Administration for ASD.^[^ ^74^, ^75^ ^]^ Aripripazole is used for ASD to alleviate irritability, hyperactivity, inappropriate language, and stereotypy;^[^ ^76^ ^]^ while risperidone reduces aggression toward others, self‐injury, challenging behavior, and rapid mood changes.^[^ ^77^ ^]^ However, both drugs have significant side effects,^[^ ^75^, ^78^ ^]^ and it is possible that treatment with MP‐10 will eliminate the need for both drugs in patients with FOXP1 syndrome.

Overall, our results strongly suggest that individuals with FOXP1 syndrome may benefit from the administration of MP‐10 or other potent PDE10A antagonists, especially if treatment is initiated at a young age. Thus, our study may be the first step toward a specific treatment for FOXP1 syndrome that not only alleviates individual symptoms but also targets two of the overarching causes of the disorder, namely the imbalance between direct and indirect pathway activity due to reduced Pde10a levels and presumed underlying neuroinflammation.

## 4 Experimental Section

### Animals

Mice were kept in a specific pathogen‐free Biomedical Animal Facility under a 12‐h light/dark cycle with ad libitum access to water and food. All procedures were conducted in strict compliance with the National Institutes of Health Guidelines for the Care and Use of Laboratory Animals and approved by the National Institute of Mental Health animal care and use committee. The day of birth was considered as postnatal day (P) 0.5.

### Generation of Nestin‐Cre (*Foxp1* ^‐/‐^) Mice

Homozygous floxed *Foxp1* mice^[^ ^79^ ^]^ were crossed with Nestin‐Cre transgenic mice (B6.Cg‐Tg(Nes‐cre)1Kln/JIn)^[^ ^80^ ^]^ heterozygous for the floxed *Foxp1* allele.

### Generation of *Foxp1* ^±^ Animals

WT female mice were crossed with male mice, heterozygous for the *Foxp1* KO allele (*Foxp1* ^±^).^[^ ^81^ ^]^

Floxed *Foxp1* mice, Nestin‐Cre deleter mice and *Foxp1* ^±^ mice were backcrossed with C57BL/6J mice for at least 12 generations to obtain congenic animals.

Animal studies were approved by the Regierungspräsidium Karlsruhe, Germany (approval number 35–9185.81/G‐105/16, 35–9185.81/G‐86/14, 35–9185.81/G‐271/17 and 35–9185.81/G‐150/22).

### Mouse Treatment

A comparable number of male and female WT and *Foxp1* ^±^ mice received a daily dose of 1.5 mg kg^−1^ MP‐10 (Merck KGaA, Darmstadt, Germany) (stock solution (20 mg mL^−1^, dissolved in DMSO) diluted with PBS) or vehicle (PBS with a corresponding amount of DMSO) by intraperitoneal injection starting immediately after birth, for a period of 29 consecutive days. To study microglia, animals were treated with the same dose of MP‐10 from birth to P8.

All treatments (Vehicle or MP‐10) were administered by the same experimenter to ensure consistency in handling and dosing. While the treatment condition was known to the experimenter, the genotype of the animals remained blinded throughout the entire study.

### cDNA Synthesis

Total RNA was prepared from frozen mouse brain tissue samples using peqGOLD TriFast (PEQLAB‐Life Science). First‐strand cDNA synthesis was performed with 1.5 µg RNA using a Superscript II reverse transcriptase kit (ThermoFisher Scientific) and oligo dT_12‐18_‐primers (ThermoFisher Scientific) according to the manufacturer's instructions.

### Quantitative Real‐Time PCR

Quantitative real‐time PCR was performed using the qTOWER system (Analytic Jena) with an annealing temperature of 60 °C using SYBR Green No‐ROX Fast Mix (Bioline) according to the manufacturer's instructions. Each of the samples was analyzed in triplicate and relative mRNA levels were assessed using the Standard Curve Method by normalization to succinate dehydrogenase complex subunit A (*Sdha1*) and hypoxanthine phosphoribosyltransferase 1 (*Hprt1*). All primer sequences were listed in Table S2 (Supporting Information).

### nCounter Analysis

Expression analysis was performed from 25 ng total RNA from striatal tissue using the nCounter system Gene 1 (NanoString Technologies, Seattle, WA, USA). The nCounter Neuroinflammation Panel (XT‐CSO‐MNROI1‐12) including site‐specific markers was hybridized as recommended by the manufacturer. Background correction and normalization of data were performed via NanoStringsoftware nSolver 4.0 (NanoString Technologies). Stably expressed reference genes were chosen for normalization based on the geNorm method (see Table S1, Supporting Information).

### Protein Analysis

Protein isolation was performed using standard protocols. Western blot analysis was executed using the Odyssey Infrared Imaging System (LI‐COR Biosciences, Lincoln, NE, USA). The following primary antibodies were used: rabbit anti‐Foxp1 (Abcam, Cambridge, UK), rabbit anti‐Pde10a (Abcam, Cambridge, UK), and mouse anti‐GAPDH (Abcam, Cambridge, UK). IRDye 800CW and IRDye 680 (LI‐COR Biosciences, Lincoln, NE, USA) were used as secondary antibodies according to the manufacturer's instructions. Protein bands were quantified using Image Studio Lite 3.1 software (LI‐COR Biosciences).

### cAMP Measurement

Frozen striatal tissue samples were processed using a modified protocol for the extraction of energy metabolites.^[^ ^82^ ^]^ Briefly, frozen murine striata were homogenized on ice with precooled steel beads and 500 µL extraction buffer (acetonitrile:methanol:15 mm ammonium acetate in H₂O, 3:1:1, pH 10) using a Retsch Mill MM400 (1  ×  30 s, 25 Hz). After centrifugation (15 min, 13000 g, 4  °C), supernatants were transferred to LC‐MS‐grade vials.

Metabolite analysis was performed using an ACQUITY I‐Class PLUS UPLC system (Waters) coupled to a QTRAP 6500+ mass spectrometer (AB SCIEX) with electrospray ionization. Separation was achieved on an ACQUITY Premier BEH Amide column (100  ×  2.1 mm, 1.7 µm; Waters) at 35  °C, using a stepwise gradient of acetonitrile/water with 5 mm ammonium acetate and 0.05% ammonium hydroxide (pH 10). Data acquisition and analysis were performed with Analyst 1.7.2 and OS 2.0.0 (AB SCIEX).

### Behavioral Testing—Pup Ultrasonic Vocalization (USV) Recordings

Litters used for ultrasonic vocalization experiments had a maximum number of nine pups. At P3.5, animals were marked by foot tattoo with nontoxic animal tattoo ink. All measurements were performed in the light period at 22 °C. P4.5, P7.5, and P12.5 pups were isolated from the mother and littermates in random order and placed in the open field (42 cm x 42 cm x 42 cm) for 5 min for USV recording, then immediately returned to the home cage. USV was recorded using an UltraSoundGate condenser microphone (CM16/CMPA, Avisoft Bioacoustics) placed 30 cm above the test arena. The microphones were connected to a computer via an Avisoft UltraSoundGate USG416H audio device. USV recordings were analyzed using SASLabPro software (Avisoft Bioacoustics) and a fast Fourier transform (FFT) was conducted (512 FFT length, 100% frame, Hamming window, 75%‐time window overlap). To exclude software errors, the calls were also analyzed manually. The USV analysis was conducted in a blinded manner without knowledge of the genotype and sex of the pups.

### Behavioral Testing—Open Field Test

The mice were placed in the center of a 40 cm × 40 cm white box with 40 cm high walls and were allowed to move freely for 10 min. The light intensity was 290 lx in the center of the room. The behavioral experiments were digitally recorded and evaluated using the ANY‐maze video tracking system. The presence of the mice in the outer 4 cm close to the wall was calculated as “time in the outer zone”.

### Behavioral Testing—Dark‐Light Box

The two‐compartment light‐dark box test (also termed the black‐white box test or light‐dark exploration test) was an ethological model of anxiety‐like behavior designed for mice, based on the aversion of rodents to white and brightly illuminated compartments. The apparatus consists of a brightly lit (400 lx) larger chamber (41 cm x 41 cm) and a smaller dark chamber (20 cm x 41 cm). At P24 the mouse was placed into the dark chamber and automatically tracked and videotaped for 10 min (Sygnis‐Tracker, Sygnis). Time spent in the light chamber, number of visits and total distance travelled were evaluated.

### Behavioral Testing—Hole Board

At P26, the mice were placed in the center of a hole board (40 cm x 40 cm x 3.5 cm) containing 16 holes (diameter: 3 cm, depth: 3.5 cm). Animals were initially allowed to explore for 10 min. Head‐dipping into the holes was automatically recorded via infrared emitters.

### Behavioral Testing—Elevated Plus Maze

The test measures the conflict between the natural tendency of mice to explore a novel environment and the aversive properties of a brightly lit open arena. The apparatus consists of a cross‐shaped platform (gray, opaque plastic material, the platform was 70 cm above the ground), with equally sized arms (W 6 cm × L 35 cm) and a central intersection (6 cm × 6 cm), allowing the animals to move freely into each zone of the maze. Two of the arms (opposite each other) were flanked by 17 cm opaque walls; the remaining two arms have no walls. Light intensities in the central zone, opened and closed arms were set to 230, 230, and 160 lux, respectively. Animals were placed in the central intersection and allowed to explore the maze for 10 min. The experiment was monitored with a digital camera and Sygnis Tracker software. The number of visits, time spent in each arm, and distance traveled in each arm were recorded. Visits of the open arm were calculated as the percentage of total arm visits, and time in the open arm was calculated as the percentage of total arm time.

### Behavioral Testing—Social Interaction

A social interaction box (Harvard Apparatus) divided into three compartments was used. The social arena consisted of a transparent box (42 × 60 cm) with two transparent sliding doors that divided the left, right, and center chambers (42 × 20 cm each). In the first 5 min session, the tested mouse was placed in the central chamber with the sliding doors open to allow access to the other two chambers for habituation. In the second session, an empty cylindrical cage and another cylindrical cage housing an unfamiliar C57BL/6N mouse of the same sex and age as the tested mouse were placed in the corners of the left and right chambers. The tested mouse was placed in the central chamber and allowed to explore the arena for 5 min. Once this session was completed, another unfamiliar C57BL/6 mouse (novel mouse) with the same sex and age as the tested mouse was put in the empty cylindrical cage. The tested mouse was then allowed to explore the arena for 5 min. In the fourth session, the mouse in the cylindrical cage from session 2 was replaced by an unfamiliar C57BL/6 mouse of the opposite sex. The tested mouse was then allowed to explore the arena for 5 min. The location of the cages was rotated between trials. The number of observed contacts of the tested mice was counted manually.

### Immunofluorescence

Mouse brains were fixed by perfusion with 4% paraformaldehyde and then dehydrated through an ethanol series and isopropanol. Brains were cleared in toluene prior to infiltration and embedded in paraffin for sectioning. 10 µm paraffin sections were prepared. Sections were deparaffinized and rehydrated through an ethanol series, followed by incubation in citrate buffer for antigen retrieval. Immunostaining was performed using standard protocols. Brain sections were incubated overnight with primary antibodies at 4 °C and incubated with appropriate fluorescent secondary antibodies for 1h at room temperature. The following primary antibodies were used: goat anti‐GFAP (Abcam, Cambridge, UK) 1:1000, rabbit anti‐Iba1 (ThermoFisher SCIENTIFIC, Dreieich, Germany) 1:1000.

### Confocal Microscopy and 3D Analysis of Microglial Morphology

For the 3D reconstruction of microglia, z‐stack images of the striatum (6‐8 images per animal) were acquired (50 µm depth, 1 µm steps, × 40 magnification) using the Nikon AX confocal (Nikon Imaging Center, Heidelberg. Imaris (10.0, Oxford Instruments) was used to reconstruct and morphologically analyze microglia as previously described.^[^ ^29^ ^]^ In brief, raw confocal files were imported into Imaris and further processed for surface reconstruction (background subtraction, surface detail 1.2 µm, largest diameter 1.2 µm). Microglia with a volume of <15 0µm^3^ or >1500 µm^3^ were filtered out before data analysis. A new masked channel was created for filament analysis, and images were processed using automated threshold detection with maximum seed point placements. Starting points were added manually to ensure that each microglia had exactly one starting point in the soma center. For microglial morphology analysis, the average values for each animal individually (surface area, volume, etc.) were calculated, and data points reflect the mean values for each morphological parameter.

Sholl Analysis was performed using Imaris in the filament reconstruction mode, and individual data sets were exported into separate Excel files for further analysis. In the respective sholl plots, each data point refers to the mean sholl intersections (average of the respective cohort, ±SEM, calculated from all microglia of each animal).

### Magnetic Resonance Imaging (MRI)

MRI was carried out at the small animal imaging core facility at the German Cancer Research Center (DKFZ), Heidelberg using a Bruker BioSpec 3Tesla (Ettlingen, Germany) with ParaVision software 360 V1.1. For the imaging, mice were anesthetized with 3.5% sevoflurane in the air. For lesion detection T2 weighted imaging was performed using a T2_TurboRARE sequence: TE = 48 ms, TR = 3350 ms, FOV 20 × 20 mm, slice thickness 1 mm, averages = 3, Scan Time 3m 21 s, echo spacing 12 ms, rare factor 8, slices 20, image size 192 × 192.

### Statistical Analysis

IBM SPSS STATISTICS 21 and Microsoft Office Excel software were used to analyze the data. Outliers in the data were determined via IBM SPSS STATISTICS 21 and excluded from further analysis. All data were checked for normal distribution via the Kolmogorov‐Smirnov and Shapiro‐Wilk test. If appropriate, two‐way ANOVA was performed using litter as a cofactor.

## Conflict of Interest

The authors declare no conflict of interest.

## Author Contributions

H.F. together with G.A.R. planned the experiments. H.F. supervised and carried out experiments and analyzed results. J.W. performed the behavioral tests and analyzed the results. N.K. was involved in the behavioral experiments. F.A. and T.S. analyzed microglia and astrocyte morphology with advice from V.G. S.S. helped with transcriptome analysis. C.P.S. supported the study. H.F. and G.A.R. wrote the paper. All coauthors commented on the paper.

## Supporting information



Supporting Information

Supporting Information

## Data Availability

The data that support the findings of this study are available in the supplementary material of this article.
