# Supplementary material for: Inhibition of Phosphodiesterase 10A by MP‐10 Rescues Behavioral Deficits and Normalizes Microglial Morphology and Synaptic Pruning in A Mouse Model of FOXP1 Syndrome
Source: Adv Sci (Weinh). 2025 Jun 26;12(36):e00623. doi: 10.1002/advs.202500623 (PMC12462984; doi:10.1002/advs.202500623)
Supplement: Supplementary file 1 — Supporting Information [file ADVS-12-e00623-s002.docx]

**Supporting Information**

Suppl. Figure S1. **Neither Untreated nor MP-10-Treated *Foxp1^+/-^* Mice Show Changes in the Social Interaction Test and the Elevated Plus Maze Test Compared to WT Animals. A,** Social Interaction test, novel mouse with same sex and with opposite sex (**B**). **C**, Elevated Plus Maze Test. For all box-and-whisker plots, the boxes represent the first and third quartiles, the whiskers represent the 95% confidence interval, and the lines within the boxes represent the median (no statistically significant differences as determined by two-way ANOVA followed by Bonferroni post hoc test).

Suppl. Figure S2. **Nestin-Cre (*Foxp1*^-/-^) Mice Show Severely Increased Anxiety Behavior and Hyperactivity. A,** Example image of the movement pattern of a WT compared to a Nestin-Cre (*Foxp1*^-/-^) mouse. **B**, Nestin-Cre (*Foxp1*^-/-^) animals travel a greater distance in the open field, spend significantly less time in the centre and enter it significantly less often than WT animals. It is obvious that the animals avoid the middle of the open field. **C**, Dark-Light-Box; Nestin-Cre (*Foxp1*^-/-^) mice visit the light box significantly less often than WT animals and spend significantly less time in it. **D**, Hole board; Nestin-Cre (*Foxp1*^-/-^) animals show reduced head dipping in holes compared to WT. For all box-and-whisker plots, the boxes represent the first and third quartiles, the whiskers are 95% confidence intervals, and the lines within the boxes are medians. Black asterisks indicate significant difference (*p ≤ 0.05, ***p ≤ 0.001, two-sided t-test).

Suppl. Figure S3. **Dysregulated Neuroinflammatory Marker Genes and Signaling Pathways in the *Foxp1*^+/-^ Striatum at P8.** nCounter analysis was performed on striatal tissue using a neuroinflammation panel with 770 targets. **A**, Relative mRNA expression of *Akt1*, *Cntnap2*, *Il10rb, Pten*, and *Gpr34* in WT and *Foxp1*^+/-^ tissue. These genes which are discussed in the manuscript are among the 111 genes that were nominally significantly altered. **B**-**C**, Network of enriched terms in *Foxp1*^+/-^ tissue: **B**, colored by cluster ID, where nodes that share the same cluster ID are typically close to each other and **C**, colored by p-value, where terms containing more genes tend to have a more significant p-value.

Suppl. Figure S4. **Adult *Foxp1*^+/-^ Mice do not Show a *Hydrocephalus e Vacuo*.** Cortical thickness and lateral ventricle size were assessed in WT and *Foxp1*^+/-^ mice aged >10 weeks by MRI (preclinical 3 Tesla small animal tomograph, ICON, Bruker, Germany). Cortical thickness was determined at three different positions (5, 6 and 7 mm behind the eye). **A**, Example MRI image of a WT and *Foxp1*^+/-^ brain. Cortical thickness (**B**) and size of lateral ventricles (**C**) normalized against brain volume.

Suppl. Figure S5. **Network of Enriched Terms in the MP-10 Treated *Foxp1*^+/-^ Striatum at P8.** nCounter analysis was performed on striatal tissue using a neuroinflammation panel with 770 targets. The Network of enriched terms in MP-10 treated *Foxp1*^+/-^ tissue: **A**, colored by cluster ID, where nodes that share the same cluster ID are typically close to each other and **B**, colored by p-value, where terms containing more genes tend to have a more significant p-value.

Suppl. Figure S6. **Striatal *Foxp1*^+/-^ Astrocytes do not Display Morphological Changes at P8.** Astrocytes and microglia were labeled on coronal cryosections of WT and *Foxp1*^+/-^ pups by fluorescent antibody staining for GFAP and Iba1, respectively, and analyzed using 3D IMARIS software. **A**, No changes in the morphology of the astrocytes (green) were observed, and their number did not differ between the genotypes. **B**, Quantification of microglia numbers showed no differences between WT and *Foxp1*^+/-^ tissue. For all box-and-whisker plots, the boxes represent the first and third quartiles, the whiskers represent the 95% confidence interval, and the lines within the boxes represent the median (no statistically significant differences, as determined by two-sided t-test).

Suppl. Figure S7. **MP-10 treatment fails to rescue altered microglial morphology in the hippocampus of *Foxp1*^+/-^ mice at P8.** WT and *Foxp1*^+/-^ animals were treated daily with either vehicle or MP-10 immediately after birth, and hippocampal microglia morphology was subsequently assessed by 3D reconstruction with Imaris at P8. Microglia were labeled by immunofluorescent antibody staining for Iba1. **A**, Coronal section showing the analyzed CA1 region of the hippocampus. **B**-**F**, Microglia from vehicle- and MP-10-treated *Foxp1*^+/-^ mice exhibit increased cell volume (B) and surface area (C), while filament length (D) and branching (E) are significantly reduced compared to vehicle-treated WT mice.
